# Screening of Chemical Composition, Antimicrobial and Antioxidant Activities in Pomegranate, Quince, and Persimmon Leaf, Peel, and Seed: Valorization of Autumn Fruits By-Products for a One Health Perspective

**DOI:** 10.3390/antibiotics12071086

**Published:** 2023-06-21

**Authors:** Vanessa Silva, Adriana Silva, Jessica Ribeiro, Alfredo Aires, Rosa Carvalho, Joana S. Amaral, Lillian Barros, Gilberto Igrejas, Patrícia Poeta

**Affiliations:** 1 Microbiology and Antibiotic Resistance Team (MicroART), Department of Veterinary Sciences, University of Trás-os-Montes and Alto Douro (UTAD), 5000-801 Vila Real, Portugal; 2 Associated Laboratory for Green Chemistry (LAQV-REQUIMTE), University NOVA of Lisbon, 2829-516 Caparica, Portugal; 3 Department of Genetics and Biotechnology, University of Trás-os-Montes and Alto Douro (UTAD), 5000-801 Vila Real, Portugal; 4 Functional Genomics and Proteomics Unit, University of Trás-os-Montes and Alto Douro (UTAD), 5000-801 Vila Real, Portugal; 5 Centro de Investigação de Montanha (CIMO), Instituto Politécnico de Bragança, Campus Santa Apolónia, 5300-253 Bragança, Portugal; jamaral@ipb.pt (J.S.A.);; 6 Centre for the Research and Technology of Agro-Environmental and Biological Sciences, CITAB, University of Trás-os-Montes e Alto Douro, 5000-801 Vila Real, Portugal; 7 Department of Agronomy, School of Agrarian and Veterinary Sciences, University of Trás-os-Montes e Alto Douro, 5000-801 Vila Real, Portugal; 8 Laboratório Associado para a Sustentabilidade e Tecnologia em Regiões de Montanha (SusTEC), Instituto Politécnico de Bragança, Campus de Santa Apolónia, 5300-253 Bragança, Portugal; 9 Associate Laboratory for Animal and Veterinary Science (AL4AnimalS), University of Trás-os-Montes and Alto Douro (UTAD), 5000-801 Vila Real, Portugal; 10 Veterinary and Animal Research Centre (CECAV), University of Trás-os-Montes and Alto Douro (UTAD), 5000-801 Vila Real, Portugal

**Keywords:** antimicrobial, antioxidant, phenolic compounds, fruits, by-products, antimicrobial resistance, pomegranate, quince, persimmon

## Abstract

Antimicrobial resistance is increasing globally and is now one of the major public health problems. Therefore, there is a need to search for new antimicrobial agents. The food industry generates large amounts of by-products that are rich in bioactive compounds, such as phenolic compounds, which are known to have several health benefits, including antioxidant and antimicrobial properties. Thus, we aimed to characterize the phenolic compounds present in pomegranate, quince, and persimmon by-products, as well as their antioxidant and antimicrobial activities. Phenolic compounds were extracted from pomegranate, quince, and persimmon leaves, seeds, and peels using a mixture of ethanol/water (80/20). The polyphenol profile of the extracts was determined by high-performance liquid chromatography. The antioxidant activity of the extracts was determined by the 2,2-diphenyl-1-picrylhydrazyl (DPPH), ferric reducing antioxidant power (FRAP), and cupric reducing antioxidant capacity (CUPRAC) methods. Antimicrobial susceptibility was evaluated using the Kirby–Bauer disk diffusion method. In general, leaves showed higher concentrations of phenolics than the peel and seeds of fruits. In total, 23 phenolic compounds were identified and quantified, with sanguiin and apigenin-3-*O*-galactoside being present in the highest concentrations. Leaf extracts of pomegranate showed higher antioxidant activities than the other components in all methods used. In general, all extracts had a greater antimicrobial activity against Gram-positive bacteria. Persimmon leaf and seed extracts inhibited a greater number of bacteria, both Gram-positive and -negative. The lowest minimum inhibitory concentration (MIC) detected among Gram-positive and -negative bacteria was 10 mg/mL for pomegranate peel and leaf extracts against *Staphylococcus aureus* and *S. pseudintermedius* and for pomegranate leaf extract against *Escherichia coli*. Our results reinforce the need to value food industry by-products that could be used as food preservatives and antibiotic adjuvants against multiresistant bacteria.

## 1. Introduction

The generation of food waste is a worldwide challenge and concern since it is related with negative environmental impact, underutilization of resources, and tangible losses of economic value [1,2,3]. Food waste occurs throughout the entire food supply chain by producers, retailers, and/or ultimate consumers. Fruit injuries, bruising, and over-ripening during food transportation and storage are some of the most common reasons that lead to food waste. This food is rejected because most consumers are reluctant to choose imperfect foods in terms of shape, color, size, appearance, and freshness [4]. It has already been described that food waste and by-products, such as peels, seeds, shells, pomace, and leaves are rich in bioactive compounds, fibers, enzymes, and antioxidants, which make them useful and interesting for the production of functional foods and drugs and also because of their potential for the cosmetic industry [5]. Moreover, natural products are valuable and show unquestionable therapeutic properties linked to low toxicity and high efficiency [6]. In this way, the transformation of food-industry waste into value-added products supports the concept of a circular economy. 

Among numerous compounds that present bioactivity, phenolic compounds are the ones that have drawn the attention of researchers and industry [7]. These compounds, also named polyphenols, are a result of the secondary metabolism of plants and can often be found in foods that are consumed daily by humans [5]. Polyphenols have an important role regarding protection, chemical defenses, and as pollinator attractants [8,9]. They have also demonstrated to be valuable against several human diseases, including cardiovascular disease, cancer, and diabetes [10]. It has been described that they can perform as antimicrobial, antioxidant, anti-inflammatory, anti-hepatotoxic, antidiarrheal, antiviral, anti-ulcer, and antiallergic agents [7]. Their mechanisms of action are not fully elucidated, but studies have identified several techniques, such as rupturing the outer cell membrane, modifying the structure and function of the cytoplasmic membrane, complexation with the cell wall, substrate withdrawal, interacting with genetic material, enzymatic inactivation, disrupting proton and electron flow, and inhibiting active transport. However, it is believed that the most common mechanism of antimicrobial action of phenolic compounds is through their interaction with the cell membrane [11]. 

The antimicrobial properties of some polyphenols have been accountable for capturing the attention of researchers due to the rising increase of drug-resistant bacteria detected in the last years [7]. For several years, people were powerless in the face of different epidemics, such as smallpox, malaria, syphilis, tuberculosis, and cholera, among many others infectious diseases. The discovery of antibiotics along with the extensive research focused on pathogens began to improve this condition [10]. Antibiotics allowed us not only to treat bacterial infections but also to improve quality of life and life expectancy [12]. Since then, the consumption of antibiotics has significantly increased and, unfortunately, the excessive and inappropriate use of this class of molecules has led to the development of mechanisms that enable bacteria to resist antimicrobial agents [13]. Consequently, the effectiveness of the drugs that are now available to treat bacterial infections has been severely affected [14]. Antibiotic resistance is one of the world’s most pressing health issues, and it has been estimated that by 2050, it will be the cause of death for 10 million people [15]. Given this alarming scenario, new alternative sources of compounds that present antimicrobial activity, compounds with antibiotic resistance-modulatory properties, and low-cost sources of natural antioxidant compounds are necessary [7].

The One Health concept recognizes the interconnection between human, animal, and environmental health, and seeks integrated solutions to promote the health of all living beings. In the case of food industry by-products, there are several ways to approach their sustainable management and use in the context of One Health, such as waste reduction, which can be done through more efficient production practices, better packaging and logistics strategies, and consumer awareness; recycling and reusing food industry by-products instead of discarding them; valorization of by-products since they often contain valuable nutrients and compounds with benefits for human and animal health; and proper management of by-products to avoid risks to animal and human health, including monitoring residues and contaminants, implementing good hygiene and food safety practices, and adopting measures to control diseases that may arise from these by-products. In short, integrating the problem and the use of food industry by-products in the context of One Health involves a holistic and collaborative approach, which considers the impacts on human, animal, and environmental health, and seeks sustainable and beneficial solutions for all parties involved.

Bearing this in mind, one economical and feasible approach to reduce the food industry impact and fight antimicrobial resistance is to extract bioactive compounds from food waste and by-products and use them as potential antibiotic adjuvants. Therefore, the aim of this work is to extract phenolic compounds from different components of three fruits (pomegranate, persimmon, and quince), characterize their content of phenolic compounds, and evaluate their antioxidant and antimicrobial activity. This work provides information for the development of new antimicrobial agents while promoting a sustainable methodology.

## 2. Results and Discussion

### 2.1. Phenolic Compounds

Pomegranate, quince, and persimmon peels, seeds, and leaves are considered as waste; nevertheless, they have received much attention recently due to their chemical content of bioactive compounds. The total phenolic content (TPC) of each extract was obtained by the Folin–Ciocalteu assay. The results for total phenolic content of the pomegranate, quince, and persimmon by-products are presented in Table 1. In general, the leaves showed higher concentrations of phenolics than the peel and seeds of fruits. When comparing the three fruits, pomegranate leaf extract had the highest concentration of phenolics (333.02 ± 21.22 µg/mg), followed by the quince leaf extract (209.78 ± 14.28 µg/mg). However, most studies that evaluated the TPC of both pomegranate leaves and peels reported that the peel extracts had higher content of phenolics than the leaf extracts [16,17,18]. It is well known that TPC may be influenced by the growing conditions, as several factors such as light, temperature, and nutrients in the soil may affect the composition of the fruit [19]. Other studies focused only on the TPC of pomegranate peel and seeds and showed that regardless of the type of solvent or cultivar used, peel extracts had a higher TPC than seeds, which is in accordance with our results [20,21,22]. In our study, the seeds were the component with the lowest concentration of phenolic compounds in both quince (12.54 ± 1.09 µg/mg) and pomegranate extracts (21.01 ± 1.19 µg/mg). However, surprisingly, the persimmon seed extract had higher content of phenolics (148.17 ± 5.92 µg/mg) than the peel and seeds of all fruits. Our results are in accordance with the results of Jang et al. (2010), who reported a higher TPC in seeds (81.81 mg/g) than in peels (6.92 mg/g) despite the fact that in our study we obtained much higher TPC values for both components [23]. However, most studies focus only on one individual component of persimmon. One study reported a very low TPC in persimmon seeds of four cultivars when compared to our results [24]. Other studies reported a lower TPC in persimmon leaves and peels when compared to our results. Hossain et al. (2018) studied the TPC in leaves of different persimmon cultivars and reported a concentration ranging from around 60 to 112 mg/g [25]. Choe et al. tested the influence of different concentrations of ethanol on the extraction of phenolics from persimmon peels and reported a TPC of 12.39 mg/g for 70% ethanol [26]. Finally, regarding the quince extracts, the leaves had the highest TPC (209.78 µg/mg) of the three quince components, whereas the seed extract had the lowest TPC (12.54 ± 1.09 µg/mg) of all components studied in this work. These results are in accordance with other studies, although we obtained higher values than most of these studies [27,28,29]. Regarding the quince peel, Stojanović et al. reported a concentration of TPC much higher than in our study (ranging from 140.12 µg/g to 202.92 µg/g), which may be explained by the different extraction method used [19]. However, considerably lower content (11.9 mg/g) was obtained by Tzanakis et al. [30].

In order to obtain the qualitative and quantitative profile of the chemical composition of the extracts, they were further evaluated by high-performance liquid chromatography with diode array detection (HPLC-DAD). It was only possible to identify phenolic compounds in the extracts of pomegranate leaf and peel, persimmon, and quince leaf and peel. The results of the phenolic composition of the extracts are shown in Table 2. Overall, 23 different compounds were identified and quantified: 13 compounds in the pomegranate leaf (9 hydrolyzable tannins, 3 flavones, and 1 hydroxycinnamic acid); 5 in the pomegranate peel (hydrolyzable tannins); 5 in the persimmon peel (1 hydroxyflavonoid and 4 flavonols); 10 in the quince leaf (5 catechins, 3 flavonols, 1 quinic acid, and 1 hydroxycinnamic acid); and 8 in the quince peel (4 catechins, 2 flavonols, 1 quinic acid, and 1 hydroxycinnamic acid). Pomegranate extracts are known to be rich in different types of ellagitannins including punicalagin, punicalin, and ellagic acid found in different matrixes such as pomegranate juice, husk, and peel [31]. Ellagitannins are a class of bioactive compounds mainly composed of hydrolyzed tannins that are commonly found in pomegranates, berries, ground elm, tea, walnuts, and chestnuts [32,33]. Most of the health benefits associated with pomegranate have been ascribed to these phenolic compounds [34,35,36]. In our study, apigenins were the compounds present in the greatest concentration in the pomegranate leaf, followed by sanguiin. Sanguiin H6 has been reported to be associated with pomegranate, but it is the major ellagitannin in raspberries and other berries [37,38]. Oenothein B was also detected in pomegranate leaves. This compound constitutes a unique class of ellagitannins and has been reported to exhibit a variety of physiological activities beneficial to human health [39]. Other studies have found this compound in pomegranate aril extracts [40,41]. Although punicalagin is considered the main component in pomegranates, punicalagin α and β were only detected in pomegranate peel in our study [41]. In fact, punicalagin was the main component in the peel extract followed by ellagic acid. These results are concordant with the ones obtained by Fraschetti et al., who reported that pomegranate peel is rich in punicalagins and ellagic acid [33]. It has been shown that pomegranate juice and peel are rich in gallic acid and ellagic acid [21,42,43]. Regarding the quince extracts, in our study, the most abundant compound was caffeic acid followed by procyanidin and quercetin-3-*O*-rutinoside in the leaf extract, while in the peel extract, the major compound was caffeic acid followed by quercetin-3-*O*-rutinoside. Other studies have shown that quercetin-3-*O*-rutinoside and procyanidins are the main compounds in quince leaves, peel, and pulp [27,44]. The high concentrations of procyanidins in quince may explain the astringency and bitterness that are characteristic of this fruit [27]. Caffeic acid and derivates were also detected in high concentrations in quince in other studies [45,46]. Both caffeic acid and quinic acids, such as chlorogenic acid, have a high number of health benefits [47]. Finally, in the persimmon extracts, the most abundant compound was kaempferol-3-*O*-glucoside, and kaempferol derivates were also detected. Other studies have shown that kaempferol and derivates and quercetin are common compounds in persimmon [48,49,50,51]. Nevertheless, in contrast, Yaqub et al. reported that caffeic acid, *p*-coumaric acid, and ferulic acid were present in large quantities in persimmon extracts, which were not detected in our study [48].

### 2.2. Antioxidant Activity

Several studies have shown that the beneficial effects of antioxidants on human health are due to their capacity to reduce oxidative stress. Therefore, assessing the antioxidant capacity of foods and food industry by-products is important not only to ensure the quality of foods but also to promote the use of these components in human and animal health [52]. In our study, we investigated the antioxidant activity of ethanolic extracts of fruit components using the DPPH, FRAP, and CUPRAC methods.

As shown in Table 3, all individual components of pomegranate, persimmon, and quince had an effective and potent reducing power. Furthermore, an assessment was conducted to determine the relationship between the TPC and antioxidant activities measured using different methods. The findings revealed a significant and robust positive correlation among all the methods (*p* < 0.001). As expected, leaf extracts of pomegranate showed higher antioxidant activities than the other components in all methods used. These results are in accordance with other studies [16,53]. However, Amri et al. (2017) reported that pomegranate leaf extract had a higher antioxidant capacity with the DPPH method but not with the FRAP [53]. On the other hand, pomegranate seed extract had a significantly lower activity than leaf and peel extracts. Studies have shown similar but also contrary results to ours [16,17,21,53]. Studies conducted by Elfalleh et al. [17] and Tehranifar et al. [16] showed a higher antioxidant activity in pomegranate peels. Nevertheless, in most studies, seed extracts had the lowest antioxidant capacity. Leaf extracts of pomegranate had 91.6% DPPH scavenging activity, which, as stated by other authors, can be considered as a full absorption inhibition of DPPH because the 100% value cannot be reached since the color of the final solution is always yellowish in comparison with the colorless control solution [54]. Pomegranates are a good source of several phenolic compounds, including punicalagins, hydrolyzable tannins, anthocyanins, and ellagic acid, which are responsible for the antioxidant activity [55]. In our study, these compounds were detected in higher proportions in leaf than peel extracts.

Quince leaf extracts also showed the highest antioxidant activity, followed by peel and seed extracts. Although the number of studies evaluating the antioxidant activity of the quince leaf, seed, and peel are very scarce, a study conducted with 13 quince varieties, including the variety ‘Portugal’ which was the same as in our study, showed that this is one of the varieties with the highest antioxidant power [28]. In the same study, the percentage of DPPH scavenging activity in the leaf extracts (55.5%) was lower than that obtained in our study (82.81%). In contrast, the percentages of DPPH scavenging activity in the peel (26.39%) and seed (2.93%) extracts were higher than our study.

Contrary to the other fruits, the persimmon seed extract had the highest antioxidant activity in all methods. Nevertheless, the antioxidant activity between the persimmon leaf and seed extracts was not significantly different. Studies comparing the antioxidant activities of permission components are also quite scarce. Nevertheless, Jang et al. obtained similar results, with seeds showing a higher antioxidant capacity than leaves [23]. Other studies have shown that persimmon leaves are rich in antioxidants and that the seeds have a strong radical-scavenging activity [48,56,57].

While some phenolics may possess both antioxidant and antimicrobial properties, the correlation between these two activities is not always straightforward. The antioxidant activity of phenolic compounds primarily involves their ability to scavenge free radicals and inhibit oxidative stress [58]. On the other hand, the antimicrobial activity is related to their ability to disrupt microbial cell membranes, inhibit enzyme activity, or interfere with microbial DNA replication [7]. Although some phenolic compounds may exhibit both activities, it is important to note that the mechanisms and effectiveness of antioxidant and antimicrobial actions can vary depending on the specific compound and the targeted microorganism. The relationship between antioxidant and antimicrobial activities of phenolic compounds suggests a potential synergy between their protective effects against oxidative stress and their ability to combat microbial infections.

### 2.3. Antimicrobial Activity

Antimicrobial resistance is increasing worldwide and is becoming a public health concern. It has been more than a decade since a new class of antibiotics has been developed; therefore, the development of new antimicrobial agents and antibiotic adjuvants is essential. In this study, we investigated the antimicrobial activity of the phenolic extracts of individual fruit components against several different genera and species of medically important bacteria. In addition, since there was a great inhibition of *Staphylococcus aureus*, the antimicrobial activity of the extracts against ten multidrug-resistant MRSA of human and animal origin was also investigated. The results of the size of the inhibition zones of Gram-positive and -negative bacteria as well as the minimum inhibitory concentrations (MICs) of all inhibited strains are shown in Table 4 and Table 5. In general, the extracts had a greater antimicrobial activity against Gram-positive bacteria since all, except for *Enterococcus faecium*, were inhibited by at least one of the extracts. None of the extracts was able to inhibit the growth of *Klebsiella pneumoniae* and *Salmonella* spp. It has been shown that Gram-positive bacteria are more susceptible to the action of fruit phenolic compounds than Gram-negative bacteria [7,11,59,60,61]. This is due to the strong electronegativity of the outer membrane of the cell wall of Gram-negative organisms, which may lead to weaker interactions between the membrane and phenolics due to the multiple hydroxyl groups found in these compounds [61,62,63]. The TPC and the individual phenolic compounds in each extract may also influence their antimicrobial activity. Nevertheless, no significant correlation was found between the TPC and the size of the inhibition zones. Although, in general, the TPC is positively correlated with antimicrobial activity, this was not the case with pomegranate peel and leaf extracts. Leaf and peel extracts of pomegranate inhibited the growth of the same strains. Nevertheless, leaf extract produced larger inhibition zones in Gram-negative bacteria than peel extracts but with higher MICs, whereas peel extracts produced larger inhibition zones in Gram-positive bacteria with lower MICs. This result may be due to the different individual phenolics found in each extract. Apigenin was found in greater amounts in pomegranate leaf extract, followed by sanguiin. The antibacterial potential of apigenin against different pathogens was investigated in other studies, and, generally, this compound inhibits the growth of both Gram-positive and -negative bacteria [64,65,66,67,68]. In a study by Dong et al., apigenin had no inhibitory action against *S. aureus*. However, apigenin was responsible for a decrease in the *S. aureus* toxin, *α*-hemolysin, to low concentrations [64]. Other studies have shown that the main targets of apigenin may be the nucleic acid processing enzymes and cell/wall membrane [67,68]. The antimicrobial activity of pomegranate leaf extract may also be due to the presence of sanguiin. Studies have shown the antimicrobial activity of this compound, and Aguilera-Correa et al. hypothesized that its action against MRSA strains is due to MRSA DNA-gyrase inactivation originated by sanguiin H-6 [69]. However, pomegranate peel extract lacked both apigenin and sanguiin, or they were present in residual amounts, with punicalagin being the most abundant compound. In a study by Gosset-Erard et al., pure punicalagin had antimicrobial activity against *Escherichia coli*, *Pseudomonas*, and *Salmonella*, but in our study, among the Gram-negative bacteria, it only was effective against *E. coli* [70]. In our study, pomegranate peel extract was able to inhibit all *Staphylococcus*, with MICs mainly of 10 mg/mL. Xu et al. reported a good antistaphylococcal effect of punicalagin with MIC of 0.25 mg/mL, which may be attributed to the increase in potassium efflux and consequently morphological damage of the cell membrane [71]. Quince extracts inhibited a smaller number of bacteria than extracts from the other two fruits, having no activity against any of the Gram-negative bacteria. In a study by Benzarti et al., quince leaf extract had antimicrobial activity against *E. faecium*, *Streptococcus agalactiae*, and *Bacillus subtilis*, but it failed to inhibit the growth of *Salmonella*, *E. coli*, *S. aureus*, and *Staphylococcus epidermidis* [72]. In another study, quince leaf extract was able to inhibit the growth of *K. pneumoniae*, *Salmonella*, *E. coli*, and *Pseudomonas,* but in that study, much higher concentrations were used than in our study [73]. Quince peel extract showed antimicrobial activity only against four MRSA strains, one of human origin and three of animal origin, with MICs of 75 mg/mL, which is in accordance with another study which reported that the quince ethanolic leaf extract was only active against *S. aureus* [74]. These results may be due to the procedures used to prepare the phenolic extracts, which have been shown to influence the TPC amount and, consequently, the antimicrobial properties of the extracts [63]. The potential of quince leaf extract has been attributed to the presence of chlorogenic acid, which has demonstrated a high level of action against *S. aureus*, *Pseudomonas aeruginosa*, *E. coli*, and *Candida albicans* while catechins, often present in this extract, showed moderate activity [75]. In our study, both quince leaf and peel extracts contained chlorogenic acid and catechins, but the compounds present in the highest concentration were caffeic acid and quercetin-3-O-galactoside, respectively. Antimicrobial activity of caffeic acid against *S. aureus* has been attributed to the presence of one or more hydroxyl groups on the phenolic ring of caffeic acid [76]. Nevertheless, the inhibitory effect of caffeic acid on other bacteria occurs mainly by inhibition of enzyme activity, changing the membrane permeability and damage of the structure of proteins and DNA [77]. Finally, although the MICs of persimmon extracts were generally higher than the other fruit extracts, they were effective against a greater number of bacterial strains. Moreover, the persimmon seed extract was effective against a high number of strains while the seed extracts of pomegranate and quince had no inhibitory effect on any of the strains tested. In the study of Amri et al., persimmon extracts showed inhibitory action against *S. aureus*, *S. epidermidis*, *E. coli*, *Salmonella*, and *P. aeruginosa*, among others, but the MICs were much higher than in our study (*S. aureus*: 312.5 mg/mL; *E. coli*: 1250 mg/mL; *Salmonella*: 1250 mg/mL; and *P. aeruginosa*: 625 mg/mL) [51]. In the same study, persimmon extracts did not show antimicrobial activity against the multidrug-resistant strains, which contrasts with our results, as all of the MRSA strains used in our study were multidrug-resistant. In another study, persimmon peel and leaf extracts were also effective in inhibiting the growth of *Salmonella*, *S. aureus*, and *E. coli* [78]. However, in our study, persimmon extracts were not active against *Salmonella*. In fact, none of the extracts tested had any inhibitory effect on the growth of *Salmonella*, *K. pneumoniae*, *Listeria monocytogenes*, and *E. faecium*. In our study, kaempferol, particularly kaempferol-3-*O*-glucoside, was the most abundant compound in persimmon extracts. In the study of Fu et al. (2016), several phenolic compounds were analyzed individually regarding their antimicrobial activity, and kaempferol showed inhibitory effects against several bacterial strains, including both Gram-positive and Gram-negative strains [50]. The inhibitory effect detected with all three persimmon extracts may be due to the generation of hydrogen peroxide in persimmon tissues, since Arakawa et al. showed that the antimicrobial activity of persimmon extracts correlated with bacterial susceptibility to hydrogen peroxide [79]. The antimicrobial activity of the extracts tested in our study involves many mechanisms that may be attributed to the different phenolic compounds found in each extract and may include inhibition of DNA gyrase and nucleic acid synthesis, pore formation, and alteration of the cell membrane/wall, among others [50].

## 3. Materials and Methods

### 3.1. Plant Material and Extract Preparation

Plant material used included the individual components of three autumn fruits: pomegranate (*Punica granatum*, Acco variety), persimmon (*Diospyros kaki*, Fau Fau variety), and quince (*Cydonia oblonga*, Portugal variety), namely, leaves, peel, and seeds. Samples were collected during autumn 2020 in the north of Portugal. The individual components were manually separated, lyophilized, and mill-powdered. For the preparation of the extracts, 2 g of the lyophilized powdered samples was extracted with 100 mL of a mixture of ethanol/water (80/20), by stirring for 2 h at ambient temperature, followed by sonication for 15 min. Then, each sample was centrifugated (11,000× *g*, 15 min) and the pellet was re-extracted (Model 2100, Kubota, Japan). The supernatant was collected to undergo the centrifugation process again, with the aim of obtaining solutions with maximum purity in terms of phenolic compound content. The supernatants were collected, and the solvents evaporated under vacuum on a rotary evaporator at 40 °C. Finally, the dry residues obtained were redissolved with dimethylsulfoxide (DMSO). The extracts were stored at −20 °C until further analysis.

### 3.2. Determination of Total Phenolic Content

The total phenolic content of the extracts was evaluated by the Folin–Ciocalteu assay. The external calibration was done using different concentrations (0.01, 0.005, 0.05, 0.1, and 0.25 mg/L) of gallic acid. Briefly, 2.0 mL of solution A (mix 10 mL of 2% Na_2_CO_3_ with 0.1 mL of CuSO_4_ and 0.1 mL of sodium and potassium tartrate) was added to 200 µL of the extracts at a concentration of 20 mg/mL. The mixture was mixed, and after 4 min, 0.4 mL of 0.5 M sodium hydroxide was added. The absorbance was measured at 760 nm. The results of TPC are expressed as mg gallic acid equivalents (GAE)/g sample [80].

### 3.3. HPLC-DAD Analysis

The evaluation of the phenolic composition was performed after the redissolution of extracts to a final concentration of 5 mg/mL and filtering through a 0.22 μm filter using a solid–liquid extraction followed by high-performance liquid chromatography (HPLC) (Gilson, Villers-le-bel, France)–diode-array detector (DAD) (Thermo Electron, San Jose, CA, USA). The compound separation was performed by gradient elution on a C18 column (5 μm particle size; 250 mm × 4.6 mm) (ACE, Aberdeen, Scotland) using a mobile phase of 0.1% (*v*/*v*) trifluoroacetic acid (TFA) in water (eluent A) and 0.1% TFA in acetonitrile (eluent B) and a flow rate of 1 mL min^−1^. The chromatograms were recorded at 254, 320, and 370 nm. Phenolic compounds were identified based on retention times, UV spectra, and UV max absorbance bands with the available reference compounds. Compound quantification in the extracts was performed by the internal standard method, and the results are expressed in mg g^−1^ dry weight [81].

### 3.4. Determination of Antioxidant Activity

Antioxidant activity was determined using three colorimetric methods: the inhibition of free radicals of DPPH (2,2-diphenyl-1-picrylhydrazyl); the FRAP method (ferric reducing antioxidant power), based on the ability of antioxidants to reduce Fe^3+^ to Fe^2+^ in the presence of 2,4,6-tri(2-pyridyl)-s-triazine (TPTZ); and the CUPRAC method (cupric reducing antioxidant capacity).

#### 3.4.1. DPPH

To determine the inhibition of DPPH radicals, the method of Siddhraju and Becker (2003) was followed with some modifications [82]. This method consists of reducing the valence electron of the free hydrogen atom in DPPH by the action of antioxidants (H atom donors), resulting in the formation of hydrazine (DPPH-H). Briefly, 4 mg of DPPH was mixed with 100 mL of 95% ethanol. Then, 285 μL of DPPH solution and 15 μL of extract were added to each well of the microplate and a standard blank solution was prepared (DPPH solution and extraction solvent instead of sample). The microplates were placed in the dark and at room temperature for 30 min. After the incubation period, the absorbance was measured at 517 nm.

The % DPPH radical scavenging capacity was calculated using the following formula:

(1) $$\%\mathrm{AA}=\left(\frac{\mathrm{Abs}\;\mathrm{Blank}-\mathrm{Abs}\;\mathrm{Sample}\;}{\mathrm{Abs}\;\mathrm{Blank}}\right)\times 100$$

The result is expressed as % DPPH radical inhibition.

#### 3.4.2. FRAP

The ferric reducing antioxidant power (FRAP) method was performed according to the method of Stratil et al. [83]. This method entails the reduction of a ferric complex, 2,4,6-tripyridyl-s-triazine (Fe^3+^-TPTZ), by antioxidants to the ferric form (Fe^2+^-TPTZ). A buffer solution of acetate (300 mM, pH 3.6), TPTZ (2,4,6-tripyridyl-S-triazine) solution at 10 mM in 40 mM of HCl, and a solution of FeCl_3_.6H_2_O at 20 mM was prepared. An aqueous solution of iron sulfate was prepared to create a calibration curve with concentrations ranging from 0.0 and 1000 μM. Then, 25 μL of each sample was added to 275 μL of FRAP reagent in each well of the microplate, and the microplate was incubated at room temperature in the dark for 5 min. After this procedure, the absorbance was measured at 593 nm and reported in µmol FeSO_4_ equivalents g^−1^ dry weight.

#### 3.4.3. CUPRAC

To quantify the cupric reducing antioxidant capacity (CUPRAC), the method of Apak et al. was used with some modifications [84]. The following solutions were prepared: CuCl_2_ (10 mM in water), neocuproin (7.5 mM in 96% ethanol), and an ammonium acetate buffer solution (N_4_CH_3_CO_2_) (1 mM, pH 7.0, in water). Then, 50 μL of CuCl_2_ and 50 μL of neocuproin were added to each well of the microplate, followed by 50 μL of N_4_CH_3_CO_2_, 25 μL of each sample, and 25 μL of water, and incubated in the dark at room temperature for 30 min. Finally, the absorbance was measured at 450 nm. A calibration curve was created with trolox as standard, and the results are expressed in μM of trolox equivalents per g of sample (μM TE/g).

### 3.5. Antimicrobial Activity

#### 3.5.1. Bacterial Strains, Culture Media, and Growth Conditions

Antimicrobial susceptibility testing was performed against 12 Gram-negative strains: 3 *Escherichia coli*, 3 *Klebsiella pneumoniae*, 3 *Klebsiella oxytoca*, 2 *Pseudomonas aeruginosa,* and 1 *Salmonella* sp., and against 8 Gram-positive strains: 2 *Listeria monocytogenes*, 2 *Staphylococcus pseudintermedius*, 2 *Staphylococcus aureus*, 1 *Enterococcus faecium,* and 1 *Enterococcus faecalis*. The best results obtained after the initial screening were obtained with *S. aureus* strains. Therefore, further antimicrobial susceptibility tests were performed against other strains of *S. aureus* with different clonal lineages and origins. Thus, 10 methicillin-resistant *S. aureus* (MRSA) strains with multidrug-resistant profiles were used: 5 of human origin and 5 of animal origin [85,86]. All bacterial strains were subcultured in brain heart infusion (BHI) agar (Oxoid, Basingstoke, UK) for 24 h at 37 °C. The strains are part of the University of Trás-os-Montes and Alto Douro. Müller–Hinton (MH) agar (Oxoid, Basingstoke, UK) was used for the antimicrobial susceptibility assay.

#### 3.5.2. Antimicrobial Susceptibility Test

The antimicrobial susceptibility testing was carried out by the Kirby–Bauer disk diffusion method against Gram-negative and Gram-positive bacteria. The evaluation the growth inhibition was performed as previously described [7]. Briefly, each phenolic extract had an initial concentration of 100 mg/mL and was diluted to 75, 50, 25, and 10 mg/mL using DMSO. Then, 20 µL of each extract concentration were loaded onto sterile blank disks (6 mm diameter). After 24 h of incubation on BHI agar, colonies of each strain were suspended in tubes containing 3 mL of saline solution to a turbidity equivalent to 0.5 McFarland standard. Then, each suspension was seeded onto MH agar plates, the disks were placed on the plates, and the plates were incubated for 24 h at 37 °C. The inhibition zones were measured with a ruler and considered an indication of antibacterial activity. The minimum inhibitory concentration (MIC) was determined as the lowest concentration among those tested that effectively inhibited bacterial growth. Disks loaded with DMSO were used as negative controls. Antibiotic disks loaded with trimethoprim/sulfamethoxazole (*E. faecalis, E. faecium,* and *L. monocytogenes*), chloramphenicol *(S. aureus, S. pseudintermedius, E. coli, K. pneumoniae*, and *K. oxytoca*), and ciprofloxacin (*P. aeruginosa* and *Salmonella* spp.) were used as positive controls.

### 3.6. Statistical Analysis

The results are expressed as mean values and standard deviation (SD). Skewness and kurtosis coefficients were computed for univariate normality analysis purposes. For the TPC and antioxidant activities, one-way analysis of variance (ANOVA) followed by Tukey’s HSD test was used with *p* = 0.05. Correlations between TPC and biological activities were analyzed using Pearson’s correlation coefficients. The analyses were carried out using IBM SPSS Statistics for Mac, Version 26.0 (IBM Corp., Armonk, New York, NY, USA).

## 4. Conclusions

Our results showed that food industry by-products, such as peels, seeds, and leaves, are a rich and diverse source of phenolic compounds with high antioxidant properties and antimicrobial activity even against multidrug-resistant bacteria. Therefore, these residues, which are mainly harmful to the environment, can be used for the benefit of human and animal health. Our study confirms the activity of phenolic compounds present in the extracts of individual fruit components against both Gram-positive and -negative bacteria which may be used as food preservatives or as antibiotic adjuvants.

The reuse of by-products from the food industry plays a key role in the One Health context and in the issue of antibiotic resistance. These by-products, which are often discarded as waste, can contain valuable nutrients and bioactive compounds. By using them sustainably, we can reduce waste, promote the circular economy, and contribute to food security. Furthermore, by adopting appropriate management practices for these by-products, it is possible to avoid environmental contamination and the spread of pathogens, thus reducing the need for the indiscriminate use of antibiotics in food production. In this way, the reuse of by-products from the food industry not only benefits human and animal health, but also protects the environment and strengthens the effectiveness of antibiotics, making it an integrated and sustainable approach to face the challenges of One Health and resistance to antibiotics.

## Figures and Tables

**Table 1:** Total phenolic content of individual fruit components (mean value ± SD, *n* = 3).

Fruit	Component	Total Phenol Content *
Pomegranate	Leaf	333.02 ± 21.22 ^a^
	Peel	71.94 ± 4.73 ^b^
	Seed	21.01 ± 1.19 ^c^
Quince	Leaf	209.78 ± 14.28 ^a^
	Peel	61.88 ± 4.56 ^b^
	Seed	12.54 ± 1.09 ^c^
Persimmon	Leaf	173.98 ± 4.51 ^a^
	Peel	20.61 ± 1.72 ^b^
	Seed	148.17 ± 5.92 ^c^

* Values expressed as µg of gallic acid/mg of residue. For each group, an ANOVA was performed, with different superscript letters indicating significant differences (*p* < 0.05).

**Table 2:** Retention time (Rt), wavelengths of maximum absorption in the visible region (λ_max_), identification, and quantification (mg/g extract) of the phenolic compounds present in the extracts of individual fruit components.

Peak	Rt (min)	λ_max_ (nm)	Identification	Quantification (mg/g of Dry Extract)				
				PL	PP	Pe	QL	QP
**1**	21.94	254	Oenothein B	15.04	nd	nd	nd	nd
**2**	22.58	254	Pedunculagin-2	5.00	nd	nd	nd	nd
**3**	23.24	320	Sanguiin-isomer	5.26	nd	nd	nd	nd
**4**	23.58	320	Sanguiin-isomer	5.98	nd	nd	nd	nd
**5**	24.24	320	Sanguiin-H6	37.39	nd	nd	nd	nd
**6**	24.73	320	*p*-coumaric acid	16.00	nd	nd	nd	nd
**7**	25.10	320	Sanguiin-isomer	29.83	nd	nd	nd	nd
**8**	25.86	320	Sanguiin-isomer	40.84	nd	nd	nd	nd
**9**	26.70	320	Sanguiin-isomer	7.39	nd	nd	nd	nd
**10**	27.44	320	Ellagic acid	16.17	nd	nd	nd	nd
**11**	28.88	320	Apigenin-3-*O*-galactoside	93.73	nd	nd	nd	nd
**12**	29.34	320	Apigenin-3-*O*-rutinoside	31.08	nd	nd	nd	nd
**13**	30.46	320	Apigenin-3-*O*-rhamnoside	25.25	nd	nd	nd	nd
**14**	22.46	320	Punicalagin α	nd	5.77	nd	nd	nd
**15**	23.15	320	Punicalagin β	nd	6.73	nd	nd	nd
**16**	24.60	320	Ellagic acid-glucoside	nd	0.91	nd	nd	nd
**17**	25.31	320	Ellagic acid-pentoside	nd	0.57	nd	nd	nd
**18**	26.88	320	Ellagic acid	nd	3.31	nd	nd	nd
**19**	22.52	320	Chlorogenic acid	nd	nd	nd	2.02	1.25
**20**	23.22	320	Procyanidin	nd	nd	nd	0.26	nd
**21**	23.89	320	Caffeic acid	nd	nd	nd	8.10	3.33
**22**	24.48	320	Procyanidin	nd	nd	nd	0.32	0.35
**23**	24.91	320	Procyanidin	nd	nd	nd	1.71	0.60
**24**	25.39	320	Procyanidin	nd	nd	nd	2.50	0.39
**25**	25.80	320	Procyanidin	nd	nd	nd	0.72	0.72
**26**	26.33	320	Quercetin-3-*O*-rutinoside	nd	nd	nd	3.62	2.07
**27**	26.99	320	Quercetin -3-*O*-xiloside	nd	nd	nd	2.90	nd
**28**	27.25	320	Quercetin-3-*O*-rhamnoside	nd	nd	nd	1.38	nd
**29**	24.31	370	Cyanidin	nd	nd	0.62	nd	nd
**30**	26.95	370	Quercetin-3-*O*-galactoside	nd	nd	1.21	nd	5.98
**31**	27.20	370	Kaempferol-3-*O*-Rhamoside	nd	nd	2.57	nd	nd
**32**	28.00	370	Kaempferol-3-*O*-glucoside	nd	nd	4.84	nd	nd
**33**	28.18	370	Kaempferol-3-*O*-Rhamoside	nd	nd	1.38	nd	nd

PL: pomegranate leaf; PP: pomegranate peel; Pe: permison leaf; QL: quince leaf; QP: quince peel; nd: not detected.

**Table 3:** Antioxidant activity of individual fruit components determined by three different methods (mean value ± SD, *n* = 3).

Fruit	Component	DPPH ^1^	FRAP ^2^	CUPRAC ^3^
Pomegranate	Leaf	91.61 ± 0.34 ^a^	6448.44 ± 524.05 ^a^	3427.78 ± 91.00 ^a^
	Peel	49.15 ± 3.06 ^b^	2262.89 ± 217.01 ^b^	807.59 ± 9.63 ^b^
	Seed	7.46 ± 0.98 ^c^	583.78 ± 31.66 ^c^	148.67 ± 16.35 ^c^
Quince	Leaf	82.81 ± 3.41 ^a^	4306.78 ± 588.45 ^a^	1626.22 ± 84.11 ^a^
	Peel	24.28 ± 1.50 ^b^	1266.67 ± 107.48 ^b^	456.44 ± 19.93 ^b^
	Seed	0.21 ± 0.04 ^c^	204.61 ± 14.68 ^c^	120.74 ± 10.78 ^c^
Persimmon	Leaf	71.65 ± 3.27 ^a^	3770.67 ± 401.06 ^a^	1374.22 ± 175.43 ^a^
	Peel	7.98 ± 1.45 ^b^	550.44 ± 37.18 ^b^	153.89 ± 11.10 ^b^
	Seed	77.97 ± 2.98 ^a^	4224.56 ± 319.90 ^a^	1573.56 ± 16.25 ^a^

^1^ expressed in % of DPPH scavenging activity; ^2^ expressed in as µmol FeSO_4_ equivalents g^−1^ dry weight; ^3^ expressed in µM/L Trolox equivalents. For each group, an ANOVA was performed, with different superscript letters indicating significant differences (*p* < 0.05).

**Table 4:** Antimicrobial susceptibility (inhibition zones, mm) of Gram-positive and Gram-negative bacteria to the extracts of fruit components.

Bacterial Strain	Inhbition Zone (mm)									
	Pomegranate			Quince			Persimmon			Antibiotics *
	Leaf	Peel	Seed	Leaf	Peel	Seed	Leaf	Peel	Seed	
**Gram-negative**										
*E. coli* 1	22	17	-	-	-	-	11	-	10	23
*E. coli* 2	8	7	-	-	-	-	9	-	7	27
*E. coli* 3	-	-	-	-	-	-	9	-	-	24
*K. pneumoniae* 1	-	-	-	-	-	-	-	-	-	30
*K. pneumoniae* 2	-	-	-	-	-	-	-	-	-	27
*K. pneumoniae* 3	-	-	-	-	-	-	-	-	-	26
*K. oxytoca* 1	-	-	-	-	-	-	10	-	9	29
*K. oxytoca* 2	-	-	-	-	-	-	8	-	9	32
*K. oxytoca* 3	-	-	-	-	-	-	8	-	-	31
*P. aeruginosa* 1	-	-	-	-	-	-	9	-	10	33
*P. aeruginosa* 2	-	-	-	-	-	-	10	-	8	35
*Salmonella* spp.	-	-	-	-	-	-	-	-	-	30
**Gram-positive**										
*S. aureus* 1	17	20	-	12	-	-	11	-	9	21
*S. aureus* 2	16	20	-	11	-	-	12	9	10	22
*S. pseudintermedius* 1	10	15	-	10	-	-	8	9	9	29
*S. pseudintermedius* 2	12	17	-	11	-	-	9	-	8	26
*L. monocytogenes* 1	-	-	-	8	-	-	-	-	-	33
*L. monocytogenes* 2	-	-	-	10	-	-	-	-	-	30
*E. faecium*	-	-	-	-	-	-	-	-	-	22
*E. faecalis*	8	15	-	9	-	-	9	-	8	25
**MRSA**										
H1	12	18	-	10	-	-	10	-	10	28
H2	17	17	-	12	8	-	14	8	15	32
H3	12	17	-	10	-	-	10	7	10	27
H4	13	18	-	10	-	-	11	-	10	29
H5	15	19	-	9	-	-	12	-	10	25
A1	17	15	-	14	10	-	16	10	-	30
A2	12	20	-	13	11	-	15	10	-	32
A3	15	19	-	12	9	-	12	9	-	30
A4	14	19	-	14	-	-	16	-	-	33
A5	12	17	-	10	-	-	11	-	-	29

* Trimethoprim/sulfamethoxazole was used for *E. faecalis*, *E. faecium*, and *L. monocytogenes*; chloramphenicol for *S. aureus*, *S. pseudintermedius*, *E. coli*, *K. pneumoniae*, and *K. oxytoca*; and ciprofloxacin for *P. aeruginosa* and *Salmonella* spp. H: human origin; A: animal origin.

**Table 5:** Minimum inhibitory concentration (MIC) of all strains inhibited by the extracts of fruit components.

Bacterial Strain	MICs (mg/mL)								
	Pomegranate			Quince			Persimmon		
	Leaf	Peel	Seed	Leaf	Peel	Seed	Leaf	Peel	Seed
**Gram-negative**									
*E. coli* 1	10	25	-	-	-	-	75	-	75
*E. coli* 2	50	75	-	-	-	-	75	-	75
*E. coli* 3	-	-	-	-	-	-	75	-	-
*K. oxytoca* 1	-	-	-	-	-	-	75	-	100
*K. oxytoca* 2	-	-	-	-	-	-	75	-	50
*K. oxytoca* 3	-	-	-	-	-	-	100	-	-
*P. aeruginosa* 1	-	-	-	-	-	-	75	-	75
*P. aeruginosa* 2	-	-	-	-	-	-	75	-	75
**Gram-positive**									
*S. aureus* 1	50	10	-	50	-	-	25	-	25
*S. aureus* 2	25	10	-	25	-	-	25	75	25
*S. pseudintermedius* 1	10	10	-	25	-	-	25	75	25
*S. pseudintermedius* 2	10	25	-	50	-	-	50	-	50
*E. faecalis*									
**MRSA**									
H1	10	10	100	25	-	-	50	-	50
H2	25	10	-	10	75	-	25	75	10
H3	25	25	-	25	-	-	50	100	50
H4	25	10	-	25	-	-	50	-	50
H5	50	10	-	50	-	-	25	-	50
A1	25	10	-	10	75	-	25	25	25
A2	50	10	-	25	75	-	10	75	25
A3	50	10	-	25	75	-	25	25	50
A4	10	10	-	10	-	-	10	-	50
A5	50	10	-	25	-	-	50	-	25

H: human origin; A: animal origin.

## Data Availability

Not applicable.

## References

[1] Jagadiswaran B., Alagarasan V., Palanivelu P., Theagarajan R., Moses J.A., Anandharamakrishnan C. (2021). Valorization of food industry waste and by-products using 3D printing: A study on the development of value-added functional cookies. Future Foods.

[2] Hassan S.A., Abbas M., Zia S., Maan A.A., Khan M.K.I., Hassoun A., Shehzad A., Gattin R., Aadil R.M. (2022). An appealing review of industrial and nutraceutical applications of pistachio waste. Crit. Rev. Food Sci. Nutr..

[3] Arshad R.N., Abdul-Malek Z., Roobab U., Qureshi M.I., Khan N., Ahmad M.H., Liu Z., Aadil R.M. (2021). Effective valorization of food wastes and by-products through pulsed electric field: A systematic review. J. Food Process Eng..

[4] Hong Y., Wang Z., Barrow C.J., Dunshea F.R., Suleria H.A.R. (2021). High-throughput screening and characterization of phenolic compounds in stone fruits waste by lc-esi-qtof-ms/ms and their potential antioxidant activities. Antioxidants.

[5] Del Rio Osorio L.L., Flórez-López E., Grande-Tovar C.D. (2021). The potential of selected agri-food loss and waste to contribute to a circular economy: Applications in the food, cosmetic and pharmaceutical industries. Molecules.

[6] Milho C., Silva J., Guimarães R., Ferreira I.C.F.R., Barros L., Alves M.J. (2021). Antimicrobials from medicinal plants: An emergent strategy to control oral biofilms. Appl. Sci..

[7] Silva V., Falco V., Dias M.I., Barros L., Silva A., Capita R., Alonso-Calleja C., Amaral J.S., Igrejas G., Ferreira I.C.F.R. (2020). Evaluation of the Phenolic Profile of Castanea sativa Mill. By-Products and Their Antioxidant and Antimicrobial Activity against Multiresistant Bacteria. Antioxidants.

[8] Alqethami A., Aldhebiani A.Y. (2021). Medicinal plants used in Jeddah, Saudi Arabia: Phytochemical screening. Saudi J. Biol. Sci..

[9] Malik F., Iqbal A., Zia S., Ranjha M.M.A.N., Khalid W., Nadeem M., Selim S., Hadidi M., Moreno A., Manzoor M.F. (2023). Role and mechanism of fruit waste polyphenols in diabetes management. Open Chem..

[10] Pancu D.F., Scurtu A., Macasoi I.G., Marti D., Mioc M., Soica C., Coricovac D., Horhat D., Poenaru M., Dehelean C. (2021). Antibiotics: Conventional therapy and natural compounds with antibacterial activity-a pharmaco-toxicological screening. Antibiotics.

[11] Silva V., Singh R.K., Gomes N., Soares B.G., Silva A., Falco V., Capita R., Alonso-Calleja C., Pereira J.E., Amaral J.S. (2020). Comparative Insight upon Chitosan Solution and Chitosan Nanoparticles Application on the Phenolic Content, Antioxidant and Antimicrobial Activities of Individual Grape Components of Sousão Variety. Antioxidants.

[12] De Simeis D., Serra S. (2021). Actinomycetes: A never-ending source of bioactive compounds—An overview on antibiotics production. Antibiotics.

[13] Akbar Z., Alquwez N., Alsolais A., Thazha S.K., Ahmad M.D., Cruz J.P. (2021). Knowledge about antibiotics and antibiotic resistance among health-related students in a Saudi University. J. Infect. Dev. Ctries.

[14] Butler M.S., Paterson D.L. (2020). Antibiotics in the clinical pipeline in October 2019. J. Antibiot..

[15] Lucien M.A.B., Canarie M.F., Kilgore P.E., Jean-Denis G., Fénélon N., Pierre M., Cerpa M., Joseph G.A., Maki G., Zervos M.J. (2021). Antibiotics and antimicrobial resistance in the COVID-19 era: Perspective from resource-limited settings. Int. J. Infect. Dis..

[16] Tehranifar A., Selahvarzi Y., Kharrazi M., Bakhsh V.J. (2011). High potential of agro-industrial by-products of pomegranate (*Punica granatum* L.) as the powerful antifungal and antioxidant substances. Ind. Crops Prod..

[17] Elfalleh W., Hannachi H., Tlili N., Yahia Y., Nasri N., Ferchichi A. (2012). Total phenolic contents and antioxidant activities of pomegranate peel, seed, leaf and flower. J. Med. Plants Res..

[18] Farag R.S., Abdel-Latif M.S., Emam S.S., Tawfeek L.S. (2014). Phytochemical screening and polyphenol constituents of pomegranate peels and leave juices. Agric. Soil Sci..

[19] Stojanović B.T., Mitić S.S., Stojanović G.S., Mitić M.N., Kostić D.A., Paunović D.Ɖ., Arsić B.B., Pavlović A.N. (2017). Phenolic profiles and metal ions analyses of pulp and peel of fruits and seeds of quince (*Cydonia oblonga* Mill.). Food Chem..

[20] Campos L., Seixas L., Henriques M.H.F., Peres A.M., Veloso A.C.A. (2022). Pomegranate Peels and Seeds as a Source of Phenolic Compounds: Effect of Cultivar, By-Product, and Extraction Solvent. Int. J. Food Sci..

[21] Singh R.P., Chidambara Murthy K.N., Jayaprakasha G.K. (2002). Studies on the Antioxidant Activity of Pomegranate (*Punica granatum*) Peel and Seed Extracts Using in Vitro Models. J. Agric. Food Chem..

[22] Campos L., Seixas L., Dias S., Peres A.M., Veloso A.C.A., Henriques M. (2022). Effect of Extraction Method on the Bioactive Composition, Antimicrobial Activity and Phytotoxicity of Pomegranate By-Products. Foods.

[23] Jang I.-C., Jo E.-K., Bae M.-S., Lee H.-J., Jeon G.-I., Park E., Yuk H.-G., Ahn G.-H., Lee S.-C. (2010). Antioxidant and antigenotoxic activities of different parts of persimmon (*Diospyros kaki* cv. Fuyu) fruit. J. Med. Plants Res..

[24] Jang I.-C., Jo E.-K., Bae S.-M., Bae M.-S., Lee H.-J., Park E., Yuk H.-G., Ahn G.-H., Lee S.-C. (2010). Antioxidant Activity and Fatty Acid Composition of Four Different Persimmon Seeds. Food Sci. Technol. Res..

[25] Hossain A., Moon H.K., Kim J.-K. (2018). Antioxidant properties of Korean major persimmon (Diospyros kaki) leaves. Food Sci. Biotechnol..

[26] Choe J.-H., Kim H.-Y., Kim Y.-J., Yeo E.-J., Kim C.-J. (2014). Antioxidant Activity and Phenolic Content of Persimmon Peel Extracted with Different Levels of Ethanol. Int. J. Food Prop..

[27] Oliveira A.P., Pereira J.A., Andrade P.B., Valentão P., Seabra R.M., Silva B.M. (2007). Phenolic Profile of *Cydonia oblonga* Miller Leaves. J. Agric. Food Chem..

[28] Sonmez F., Sahin Z. (2022). Comparative Study of Total Phenolic Content, Antioxidant Activities, and Polyphenol Oxidase Enzyme Inhibition of Quince Leaf, Peel, and Seed Extracts. Erwerbs-Obstbau.

[29] Benzarti S., Hamdi H., Lahmayer I., Toumi W., Kerkeni A., Belkadhi K., Sebei H. (2015). Total phenolic compounds and antioxidant potential of quince (*Cydonia oblonga* Miller) leaf methanol extract. Int. J. Innov. Appl. Stud..

[30] Tzanakis E., Kalogeropoulos T.H., Tzimas S.T., Chatzilazarou A., Katsoyannos E. (2006). Phenols and antioxidant activity of apple, quince, pomegranate, bitter orange and almond-leaved pear methanolic extracts. EJ Sci. Technol..

[31] Ali O.M., Hashem Y., Bekhit A.A., Khattab S.N., Elkhodairy K.A., Freag M.S., Teleb M., Elzoghby A.O., Jafari S.M. (2019). Nanostructures of gelatin for encapsulation of food ingredients. Nanoencapsulation in the Food Industry.

[32] Chen P., Guo Z., Chen F., Wu Y., Zhou B. (2022). Recent advances and perspectives on the health benefits of urolithin B, a bioactive natural product derived from ellagitannins. Front. Pharmacol..

[33] Fraschetti C., Goci E., Nicolescu A., Cairone F., Carradori S., Filippi A., Palmieri V., Mocan A., Cesa S. (2023). Pomegranate Fruit Cracking during Maturation: From Waste to Valuable Fruits. Foods.

[34] Viuda-Martos M., Fernández-López J., Pérez-Álvarez J.A. (2010). Pomegranate and its many functional components as related to human health: A review. Compr. Rev. Food Sci. Food Saf..

[35] Ismail T., Sestili P., Akhtar S. (2012). Pomegranate peel and fruit extracts: A review of potential anti-inflammatory and anti-infective effects. J. Ethnopharmacol..

[36] García-Villalba R., Espín J.C., Aaby K., Alasalvar C., Heinonen M., Jacobs G., Voorspoels S., Koivumäki T., Kroon P.A., Pelvan E. (2015). Validated Method for the Characterization and Quantification of Extractable and Nonextractable Ellagitannins after Acid Hydrolysis in Pomegranate Fruits, Juices, and Extracts. J. Agric. Food Chem..

[37] Kashchenko N.I., Olennikov D.N., Chirikova N.K. (2021). Metabolites of Siberian Raspberries: LC-MS Profile, Seasonal Variation, Antioxidant Activity and, Thermal Stability of Rubus matsumuranus Phenolome. Plants.

[38] Pachuau L. (2022). Phytochemical and Pharmacological Profile of Rubus idaeus. Bioactives and Pharmacology of Medicinal Plants.

[39] Yoshida T., Yoshimura M., Amakura Y. (2018). Chemical and Biological Significance of Oenothein B and Related Ellagitannin Oligomers with Macrocyclic Structure. Molecules.

[40] Ito H., Li P., Koreishi M., Nagatomo A., Nishida N., Yoshida T. (2014). Ellagitannin oligomers and a neolignan from pomegranate arils and their inhibitory effects on the formation of advanced glycation end products. Food Chem..

[41] Kawakami K., Li P., Uraji M., Hatanaka T., Ito H. (2014). Inhibitory Effects of Pomegranate Extracts on Recombinant Human Maltase–Glucoamylase. J. Food Sci..

[42] Mosele J.I., Macià A., Romero M.-P., Motilva M.-J., Rubió L. (2015). Application of in vitro gastrointestinal digestion and colonic fermentation models to pomegranate products (juice, pulp and peel extract) to study the stability and catabolism of phenolic compounds. J. Funct. Foods.

[43] Cheshomi H., Bahrami A.R., Rafatpanah H., Matin M.M. (2022). The effects of ellagic acid and other pomegranate (*Punica granatum* L.) derivatives on human gastric cancer AGS cells. Hum. Exp. Toxicol..

[44] Khoubnasabjafari M., Jouyban A. (2011). A review of phytochemistry and bioactivity of quince (*Cydonia oblonga* Mill.). J. Med. Plants Res..

[45] Sut S., Dall’Acqua S., Poloniato G., Maggi F., Malagoli M. (2019). Preliminary evaluation of quince (*Cydonia oblonga* Mill.) fruit as extraction source of antioxidant phytoconstituents for nutraceutical and functional food applications. J. Sci. Food Agric..

[46] Herrera-Rocha K.M., Rocha-Guzmán N.E., Gallegos-Infante J.A., González-Laredo R.F., Larrosa-Pérez M., Moreno-Jiménez M.R. (2022). Phenolic Acids and Flavonoids in Acetonic Extract from Quince (*Cydonia oblonga* Mill.): Nutraceuticals with Antioxidant and Anti-Inflammatory Potential. Molecules.

[47] Maghsoudlou Y., Asghari Ghajari M., Tavasoli S. (2019). Effects of heat treatment on the phenolic compounds and antioxidant capacity of quince fruit and its tisane’s sensory properties. J. Food Sci. Technol..

[48] Yaqub S., Farooq U., Shafi A., Akram K., Murtaza M.A., Kausar T., Siddique F. (2016). Chemistry and Functionality of Bioactive Compounds Present in Persimmon. J. Chem..

[49] Kim Y.-M., Abas F., Park Y.-S., Park Y.-K., Ham K.-S., Kang S.-G., Lubinska-Szczygeł M., Ezra A., Gorinstein S. (2021). Bioactivities of Phenolic Compounds from Kiwifruit and Persimmon. Molecules.

[50] Fu L., Lu W., Zhou X. (2016). Phenolic Compounds and In Vitro Antibacterial and Antioxidant Activities of Three Tropic Fruits: Persimmon, Guava, and Sweetsop. Biomed Res. Int..

[51] Heras R.M.-L., Quifer-Rada P., Andrés A., Lamuela-Raventós R. (2016). Polyphenolic profile of persimmon leaves by high resolution mass spectrometry (LC-ESI-LTQ-Orbitrap-MS). J. Funct. Foods.

[52] Munteanu I.G., Apetrei C. (2021). Analytical Methods Used in Determining Antioxidant Activity: A Review. Int. J. Mol. Sci..

[53] Amri Z., Zaouay F., Lazreg-Aref H., Soltana H., Mneri A., Mars M., Hammami M. (2017). Phytochemical content, Fatty acids composition and antioxidant potential of different pomegranate parts: Comparison between edible and non edible varieties grown in Tunisia. Int. J. Biol. Macromol..

[54] Miliauskas G., Venskutonis P.R., van Beek T.A. (2004). Screening of radical scavenging activity of some medicinal and aromatic plant extracts. Food Chem..

[55] Nuncio-Jáuregui N., Calín-Sánchez Á., Vázquez-Araújo L., Pérez-López A.J., Frutos-Fernández M.J., Carbonell-Barrachina Á.A., Preedy V. (2015). Chapter 76—Processing Pomegranates for Juice and Impact on Bioactive Components.

[56] Ahn H.S., Jeon T.I., Lee J.Y., Hwang S.G., Lim Y., Park D.K. (2002). Antioxidative activity of persimmon and grape seed extract: In vitro and in vivo. Nutr. Res..

[57] Steinmetz K.A., Potter J.D. (1996). Vegetables, fruit, and cancer prevention: A review. J. Am. Diet. Assoc..

[58] Akbari B., Baghaei-Yazdi N., Bahmaie M., Mahdavi Abhari F. (2022). The role of plant-derived natural antioxidants in reduction of oxidative stress. BioFactors.

[59] Singh J.P., Kaur A., Singh N., Nim L., Shevkani K., Kaur H., Arora D.S. (2016). In vitro antioxidant and antimicrobial properties of jambolan (*Syzygium cumini*) fruit polyphenols. LWT—Food Sci. Technol..

[60] Coman M.M., Oancea A.M., Verdenelli M.C., Cecchini C., Bahrim G.E., Orpianesi C., Cresci A., Silvi S. (2018). Polyphenol content and in vitro evaluation of antioxidant, antimicrobial and prebiotic properties of red fruit extracts. Eur. Food Res. Technol..

[61] Wafa B.A., Makni M., Ammar S., Khannous L., Hassana A.B., Bouaziz M., Es-Safi N.E., Gdoura R. (2017). Antimicrobial effect of the Tunisian Nana variety *Punica granatum* L. extracts against Salmonella enterica (serovars Kentucky and Enteritidis) isolated from chicken meat and phenolic composition of its peel extract. Int. J. Food Microbiol..

[62] Li A.-N., Li S., Zhang Y.-J., Xu X.-R., Chen Y.-M., Li H.-B. (2014). Resources and biological activities of natural polyphenols. Nutrients.

[63] Lima M.C., Paiva de Sousa C., Fernandez-Prada C., Harel J., Dubreuil J.D., de Souza E.L. (2019). A review of the current evidence of fruit phenolic compounds as potential antimicrobials against pathogenic bacteria. Microb. Pathog..

[64] Dong J., Qiu J., Wang J., Li H., Dai X., Zhang Y., Wang X., Tan W., Niu X., Deng X. (2013). Apigenin alleviates the symptoms of *Staphylococcus aureus* pneumonia by inhibiting the production of alpha-hemolysin. FEMS Microbiol. Lett..

[65] Özçelik B., Kartal M., Orhan I. (2011). Cytotoxicity, antiviral and antimicrobial activities of alkaloids, flavonoids, and phenolic acids. Pharm. Biol..

[66] Wang M., Firrman J., Liu L., Yam K. (2019). A Review on Flavonoid Apigenin: Dietary Intake, ADME, Antimicrobial Effects, and Interactions with Human Gut Microbiota. Biomed Res. Int..

[67] Wang M., Firrman J., Zhang L., Arango-Argoty G., Tomasula P., Liu L., Xiao W., Yam K. (2017). Apigenin impacts the growth of the gut microbiota and alters the gene expression of Enterococcus. Molecules.

[68] Wu D., Kong Y., Han C., Chen J., Hu L., Jiang H., Shen X. (2008). D-Alanine: D-alanine ligase as a new target for the flavonoids quercetin and apigenin. Int. J. Antimicrob. Agents.

[69] Aguilera-Correa J.J., Fernández-López S., Cuñas-Figueroa I.D., Pérez-Rial S., Alakomi H.-L., Nohynek L., Oksman-Caldentey K.-M., Salminen J.-P., Esteban J., Cuadros J. (2021). Sanguiin H-6 Fractionated from Cloudberry (*Rubus chamaemorus*) Seeds Can Prevent the Methicillin-Resistant Staphylococcus aureus Biofilm Development during Wound Infection. Antibiotics.

[70] Gosset-Erard C., Zhao M., Lordel-Madeleine S., Ennahar S. (2021). Identification of punicalagin as the bioactive compound behind the antimicrobial activity of pomegranate (*Punica granatum* L.) peels. Food Chem..

[71] Xu Y., Shi C., Wu Q., Zheng Z., Liu P., Li G., Peng X., Xia X. (2017). Antimicrobial Activity of Punicalagin Against *Staphylococcus aureus* and Its Effect on Biofilm Formation. Foodborne Pathog. Dis..

[72] Benzarti S., Belkadhi K., Hamdi H. (2018). Biological activities of phenolics from leaves of Tunisian *Cydonia oblonga* Miller. Allelopath. J..

[73] Semnani S.N., Hajizadeh N., Alizadeh H. (2017). Antibacterial effects of aqueous and organic quince leaf extracts on gram–positive and gram–negative bacteria. Banat. J. Biotechnol..

[74] Al-Noamy N.A.F. (2020). Detection of the Inhibitory Effect of the Leaves, Seed and Fruits of Cydonia oblonga on some Gram Positive and Negative Bacteria. Rafidain J. Sci..

[75] Fattouch S., Caboni P., Coroneo V., Tuberoso C.I.G., Angioni A., Dessi S., Marzouki N., Cabras P. (2007). Antimicrobial Activity of Tunisian Quince (*Cydonia oblonga* Miller) Pulp and Peel Polyphenolic Extracts. J. Agric. Food Chem..

[76] Stojković D., Petrović J., Soković M., Glamočlija J., Kukić-Marković J., Petrović S. (2013). In situ antioxidant and antimicrobial activities of naturally occurring caffeic acid, p-coumaric acid and rutin, using food systems. J. Sci. Food Agric..

[77] Khan F., Bamunuarachchi N.I., Tabassum N., Kim Y.-M. (2021). Caffeic Acid and Its Derivatives: Antimicrobial Drugs toward Microbial Pathogens. J. Agric. Food Chem..

[78] Reddy S.H., Al Jahwari M.R.H., Al Tobi Z.M.R. (2019). A study on FTIR, Antimicrobial, Antioxidant and Hypogycaemic effect of Diospyros kaki and *Citrullus colocynthis*. Int. J. Phytomed..

[79] Arakawa H., Takasaki M., Tajima N., Fukamachi H., Igarashi T. (2014). Antibacterial Activities of Persimmon Extracts Relate with Their Hydrogen Peroxide Concentration. Biol. Pharm. Bull..

[80] Iyda J.H., Fernandes Â., Ferreira F.D., Alves M.J., Pires T.C.S.P., Barros L., Amaral J.S., Ferreira I.C.F.R. (2019). Chemical composition and bioactive properties of the wild edible plant *Raphanus raphanistrum* L.. Food Res. Int..

[81] Aires A., Carvalho R., Matos M., Carnide V., Silva A.P., Gonçalves B. (2017). Variation of chemical constituents, antioxidant activity, and endogenous plant hormones throughout different ripening stages of highbush blueberry (*Vaccinium corymbosum* L.) cultivars produced in centre of Portugal. J. Food Biochem..

[82] Siddhuraju P., Becker K. (2003). Antioxidant properties of various solvent extracts of total phenolic constituents from three different agroclimatic origins of drumstick tree (*Moringa oleifera* Lam.) leaves. J. Agric. Food Chem..

[83] Stratil P., Klejdus B., Kubáň V. (2006). Determination of total content of phenolic compounds and their antioxidant activity in vegetables evaluation of spectrophotometric methods. J. Agric. Food Chem..

[84] Apak R., Güçlü K., Özyürek M., Karademir S.E. (2004). Novel total antioxidant capacity index for dietary polyphenols and vitamins C and E, using their cupric ion reducing capability in the presence of neocuproine: CUPRAC method. J. Agric. Food Chem..

[85] Silva V., de Sousa T., Gómez P., Sabença C., Vieira-Pinto M., Capita R., Alonso-Calleja C., Torres C., Capelo J.L., Igrejas G. (2020). Livestock-Associated Methicillin-Resistant *Staphylococcus aureus* (MRSA) in Purulent Subcutaneous Lesions of Farm Rabbits. Foods.

[86] Silva V., Almeida F., Carvalho J.A., Castro A.P., Ferreira E., Manageiro V., Tejedor-Junco M.T., Caniça M., Igrejas G., Poeta P. (2020). Emergence of community-acquired methicillin-resistant *Staphylococcus aureus* EMRSA-15 clone as the predominant cause of diabetic foot ulcer infections in Portugal. Eur. J. Clin. Microbiol. Infect. Dis..

